# Sleep Duration, Sleep Quality, and the Development of Nonalcoholic Fatty Liver Disease: A Cohort Study

**DOI:** 10.14309/ctg.0000000000000417

**Published:** 2021-10-19

**Authors:** Yoo Jin Um, Yoosoo Chang, Hyun-Suk Jung, In Young Cho, Jun Ho Shin, Hocheol Shin, Sarah H. Wild, Christopher D. Byrne, Seungho Ryu

**Affiliations:** 1 Total Healthcare Center, Kangbuk Samsung Hospital, Sungkyunkwan University School of Medicine, Seoul, KR, South Korea;; 2 Center for Cohort Studies, Total Healthcare Center, Kangbuk Samsung Hospital, Sungkyunkwan University School of Medicine, Seoul, KR, South Korea;; 3 Department of Occupational and Environmental Medicine, Kangbuk Samsung Hospital, Sungkyunkwan University School of Medicine, Seoul, KR, South Korea;; 4 Department of Clinical Research Design and Evaluation, SAIHST, Sungkyunkwan University, Seoul, KR, South Korea;; 5 Department of Family Medicine, Kangbuk Samsung Hospital, Sungkyunkwan University School of Medicine, Seoul, KR, South Korea;; 6 Department of Surgery, Kangbuk Samsung Hospital, Sungkyunkwan University School of Medicine, Seoul, KR, South Korea;; 7 Usher Institute, University of Edinburgh, Edinburgh, UK;; 8 Nutrition and Metabolism, Faculty of Medicine, University of Southampton, Southampton, UK;; 9 National Institute for Health Research Southampton Biomedical Research Centre, University Hospital Southampton, Southampton, UK.

## Abstract

The longitudinal relationship between sleep duration, sleep quality, and the risk of nonalcoholic fatty liver disease (NAFLD) is unknown. We aimed to examine the association between sleep duration, sleep quality, and NAFLD development.

**METHODS:**

Using the Pittsburgh Sleep Quality Index, sleep duration and quality were evaluated for 143,306 NAFLD-free Korean adults with a mean age of 36.6 years, who were followed for an average of 4.0 years. Hepatic steatosis (HS) was assessed using ultrasonography and liver fibrosis by the fibrosis-4 index (FIB-4) or the NAFLD fibrosis score. Flexible parametric proportional hazard models were used to determine the hazard ratios (HRs) and 95% confidence intervals.

**RESULTS:**

There were 27,817 subjects with incident HS, of whom 1,471 had incident HS plus intermediate/high FIB-4. Multivariable-adjusted HRs (95% confidence intervals) for incident HS comparing sleep durations of ≤5, 6, 8, and ≥ 9 hours with 7 hours were 1.19 (1.14–1.23), 1.07 (1.04–1.10), 0.98 (0.94–1.02), and 0.95 (0.87–1.03), respectively. The corresponding HRs for incident HS plus intermediate/high FIB-4 were 1.30 (1.11–1.54), 1.14 (1.01–1.29), 1.11 (0.93–1.33), and 1.08 (0.71–1.63). The association between sleep duration and HS plus intermediate/high FIB-4 was inverse in individuals with good sleep quality but tended to be U-shaped in those with poor sleep quality. The results were similar if FIB-4 was replaced by the NAFLD fibrosis score.

**DISCUSSION:**

In young adults, short sleep duration was independently associated with an increased risk of incident NAFLD with or without intermediate/high fibrosis score, suggesting a role for inadequate sleep quantity in NAFLD risk and severity.

## INTRODUCTION

Nonalcoholic fatty liver disease (NAFLD) is one of the most prevalent liver disorders worldwide (1), with a global prevalence of approximately 25% (2). NAFLD is now considered a multisystem disease that is associated with cardiometabolic disorders, all-cause mortality, and cardiovascular disease mortality (3,4). There are currently no approved medical therapies (5), and the first-line treatment for NAFLD management is lifestyle modification (6). Thus, it is important to identify all modifiable lifestyle factors because it is plausible that improvements in each of these factors may help prevent the development of NAFLD.

We spend, on average, a one-third of our lifetime sleeping. Sleep has been reported to play a pivotal role in cardiovascular health, as well as the endocrine and immune systems (7,8). However, in recent decades, sleep duration has decreased, with a reported prevalence of short sleep duration (defined as < 6 hours) reaching more than 20% (9). A decrease in sleep duration may adversely affect insulin sensitivity and inflammatory activity (8,10) and may therefore contribute to the development of NAFLD. Epidemiological studies have also suggested that short sleep duration is closely associated with obesity, metabolic syndrome, and cardiovascular disease (11,12), all of which are also commonly observed in patients with NAFLD (13). Currently, the relationship between sleep duration and NAFLD is controversial. A meta-analysis reported a small but significant association between short sleep duration and increased risk of NAFLD (14), whereas another meta-analysis showed no significant association between sleep duration and the risk of fatty liver disease (15). However, the findings from both these meta-analyses were determined mostly based on results from cross-sectional studies. Currently, available cohort studies on this association have limitations, including small sample sizes, lack of consideration of sleep quality and changing status of sleep habits over time, and the inclusion of elderly adults who already had a high number of comorbidities, including sleep problems (16–19). Furthermore, none of the cohort studies evaluated the impact of sleep duration on the development of the more severe form of NAFLD, incident NAFLD with liver fibrosis, the most important predictor of liver and nonliver mortality (20,21).

This study aimed to evaluate the relationship between sleep duration and sleep quality and the development of incident hepatic steatosis (HS) with and without an intermediate/high probability of liver fibrosis while accounting for time-dependent measures of change in sleep duration, sleep quality, and potential confounders during the follow-up period.

## METHODS

### Study population

**Figure 1.**
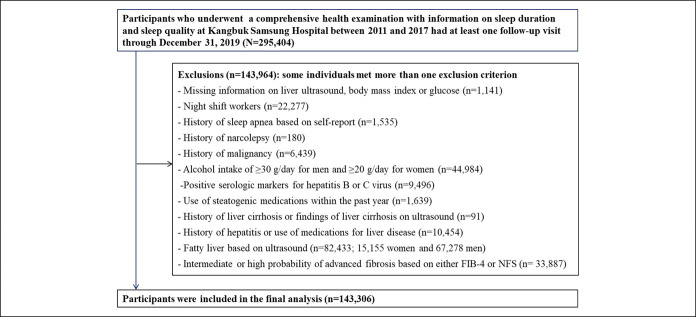
Flowchart of the included subjects.

This cohort study is a part of the Kangbuk Samsung Health Study, a cohort study of Korean adults who participated in a health examination annually or biennially at Kangbuk Samsung Hospital Total Healthcare Centers in Seoul and Suwon, South Korea, as previously described (22). This study population was restricted to individuals who underwent a health screening examination with information on sleep duration and sleep quality from March 2011 to December 2017 and had at least 1 follow-up visit by December 31, 2019 (N = 295,404). A total of 143,964 subjects met 1 or more of the exclusion criteria at baseline (Figure 1). The final sample included 143,306 subjects in the analysis. This study was approved by the Institutional Review Board of Kangbuk Samsung Hospital (2021-01-024) and was conducted in accordance with the Declaration of Helsinki. The requirement for informed consent was waived because of the use of a pre-existing deidentified data set that was routinely collected during the health screening process.

### Data collection

Data regarding the patient's demographic characteristics, behavioral factors, and medical history were collected using a standardized, self-administered questionnaire. Anthropometry, blood pressure, and serum biochemical parameters were measured by trained staff during the health examinations.

Sleep duration and quality were assessed using the validated Pittsburgh Sleep Quality Index (PSQI), 19-item self-administered questionnaire, at baseline and during the follow-up sessions (23) (see Supplementary Data, Supplementary Digital Content 1, http://links.lww.com/CTG/A709).

The diagnosis of HS was based on an abdominal ultrasound performed by an experienced radiologist who was blinded to the aim of this study. Two noninvasive indices of liver fibrosis, the fibrosis-4 index (FIB-4) and NAFLD fibrosis score (NFS), were used to assess severity of NAFLD (see Supplementary Data, Supplementary Digital Content 1, http://links.lww.com/CTG/A709; [24,25]).

### Statistical analysis

The primary endpoints were (i) the development of incident HS (regardless of fibrosis score) and (ii) the development of incident HS plus an intermediate/high probability of liver fibrosis. Incident HS and incident HS combined with an intermediate/high probability of liver fibrosis based on the FIB-4 or NFS were treated as separate endpoints in each model. The event detection date was defined as the earliest date of identification of HS or HS with an intermediate/high probability of liver fibrosis based on the FIB-4 score or NFS, which were analyzed separately. The person-years were calculated as the sum of the follow-up duration from baseline to the event detection date (HS or HS with fibrosis, separately) or until the final examination (before December 31, 2019), whichever occurred first. Incidence rates were calculated as the number of incident cases divided by the person-years of follow-up. Therefore, as the primary endpoint occurred at an unknown time point between the event detection date and the previous screening visit, a parametric proportional hazard model was used to account for this type of interval censoring and to estimate the hazard ratios (HRs) and 95% confidence intervals (CIs). In these models, the baseline hazard function was parameterized with restricted cubic splines in log time with 4 degrees of freedom. We assessed the proportional hazard assumption by examining graphs of estimated log (−log(survival)) vs the log of survival time graph. No violation of the assumption was found.

The risk of incident HS and incident HS combined with an intermediate/high probability of liver fibrosis was separately evaluated according to the sleep duration category (see Supplementary Data, Supplementary Digital Content 1, http://links.lww.com/CTG/A709).

Statistical analyses were performed using STATA version 16.0 (StataCorp LP, College Station, TX). All reported *P* values were 2-tailed, and a *P* value of < 0.05 was considered statistically significant.

## RESULTS

At baseline, the mean (SD) age and sleep duration of 143,306 subjects were 36.6 (6.6) years and 7.0 (1.1) hours, respectively. The prevalence of poor sleep quality was 20.1% and was inversely associated with sleep duration, with the highest prevalence seen in those with a short sleep duration of ≤5 hours (45.8%). The sleep duration categories were positively associated with being married but were inversely associated with age, male sex, current smoking, alcohol drinking, depressive symptoms, and obesity (Table 1).

**Table 1.** Baseline characteristics of participants by sleep duration

Characteristics	Overall	Sleep duration (hr)				
		≤5	6	7	8	≥9
Number	143,306	19,003	48,163	49,589	21,766	4,785
Age (yr)^a^	36.6 (6.6)	36.7 (7.0)	36.8 (6.8)	36.6 (6.5)	36.2 (6.3)	35.2 (6.1)
Men (%)	38.5	40.2	47.4	39.0	22.4	8.6
Obesity (%)	11.8	14.1	13.6	11.0	8.6	7.1
Current smoker (%)	13.3	15.8	15.8	12.9	8.0	3.8
Alcohol intake (%)^b^	25.0	27.4	29.2	24.6	17.1	12.4
Alcohol intake, g/d	6.2 (6.5)	6.6 (6.8)	6.9 (6.9)	6.1 (6.5)	4.8 (5.5)	3.8 (4.6)
Men	9.9 (7.7)	10.2 (7.9)	10.1 (7.7)	9.8 (7.7)	9.4 (7.6)	9.2 (7.4)
Women	3.7 (3.9)	3.9 (4.2)	3.9 (4.0)	3.6 (3.9)	3.4 (3.7)	3.3 (3.8)
HEPA (%)	14.1	15.5	14.3	14.1	13.1	11.5
High education (%)^c^	87.9	86.8	88.9	88.6	86.0	82.7
Married (%)	78.4	72.1	74.9	79.8	86.0	90.7
Depression (%)	11.6	19.7	11.8	9.2	9.5	11.9
Hypertension (%)	4.4	4.9	4.9	4.3	3.2	2.3
Diabetes (%)	0.7	0.8	0.8	0.7	0.6	0.6
History of CVD (%)	0.8	1.0	0.9	0.7	0.4	0.8
BMI (kg/m^2^)	21.9 (2.6)	22.1 (2.7)	22.1 (2.6)	21.8 (2.6)	21.4 (2.6)	21.0 (2.5)
Waist circumference (cm)	77.0 (7.2)	77.5 (8.1)	77.8 (8.1)	76.8 (7.9)	75.5 (7.6)	74.3 (7.2)
Systolic BP (mm Hg)^a^	104.8 (11.7)	104.9 (11.7)	105.8 (11.8)	104.9 (11.8)	103.0 (11.3)	101.1 (10.5)
Diastolic BP (mm Hg)^a^	66.9 (8.8)	66.9 (8.8)	67.5 (8.9)	67.0 (8.8)	65.8 (8.5)	64.7 (7.9)
Glucose (mg/dL)^a^	91.3 (9.2)	91.0 (9.7)	91.6 (9.4)	91.4 (9.2)	91.0 (8.7)	90.1 (8.7)
Total cholesterol (mg/dL)^a^	187.4 (31.6)	188.5 (31.8)	188.4 (31.5)	187.3 (31.4)	185.2 (31.6)	183.8 (32.1)
LDL-C (mg/dL)^a^	113.6 (29.4)	114.5 (29.7)	115.1 (29.6)	113.5 (29.2)	111.0 (28.8)	109.0 (28.6)
HDL-C (mg/dL)^a^	63.0 (14.9)	63.2 (15.2)	62.3 (14.9)	63.1 (14.8)	63.9 (14.8)	64.8 (14.4)
Triglycerides (mg/dL)^d^	75 (57–103)	75 (56–104)	76 (58–105)	75 (57–103)	72 (56–98)	71 (54–95)
ALT (U/L)^d^	15 (11–20)	15 (11–20)	15 (12–21)	15 (11–20)	13 (11–18)	12 (10–16)
GGT (U/L)^d^	15 (11–22)	15 (11–23)	16 (12–24)	15 (11–22)	13 (10–19)	12 (10–17)
HOMA-IR^d^	1.04 (0.71–1.48)	1.01 (0.68–1.44)	1.02 (0.69–1.46)	1.05 (0.71–1.49)	1.08 (0.73–1.53)	1.08 (0.73–1.53)
hsCRP (mg/L)^d^	0.3 (0.2–0.6)	0.3 (0.2–0.7)	0.3 (0.2–0.7)	0.3 (0.2–0.6)	0.3 (0.2–0.6)	0.3 (0.2–0.6)
Total energy intake^d,e^	1,445.1 (1,085.2–1833.5)	1,451.5 (1,072.1–1863.9)	1,456.0 (1,096.8–1851.7)	1,444.8 (1,094.1–1822.6)	1,423.3 (1,069.4–1801.3)	1,397.0 (1,017.5–1802.3)
Poor sleep quality (%)	20.1	45.8	21.8	13.2	10.8	13.4

ALT, alanine aminotransferase; BMI, body mass index; BP, blood pressure; CVD, cardiovascular disease; HDL-C, high-density lipoprotein cholesterol; HEPA, health-enhancing physical activity; hsCRP, high-sensitivity C-reactive protein; HOMA-IR, homeostasis model assessment of insulin resistance.

Data are expressed as follows:

a Mean (SD);

b ≥10 g of ethanol per d;

c ≥College graduate; and

d Median (interquartile range) or percentage.

e Among 143,306 subjects with plausible estimated energy intake levels (within 3 SDs from the log-transformed mean energy intake).

**Figure 2.**
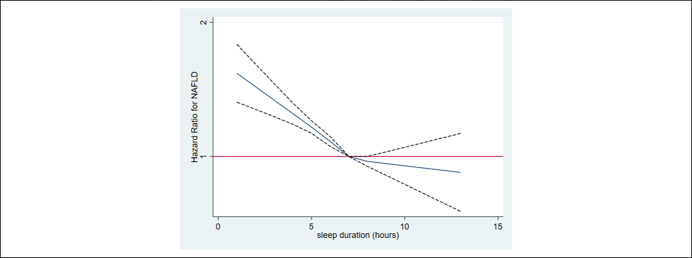
Multivariable-adjusted HRs for NAFLD. The curves represent adjusted HRs for incident NAFLD based on restricted cubic splines with knots at the 5th, 27.5th, 50th, 72.5th, and 95th percentiles of sleep duration distribution. The model was adjusted for age, sex, center, year of the screening examination, body mass index, alcohol consumption, smoking, physical activity, total energy intake, marital status, season, education level, depression, history of diabetes, and history of hypertension. HR, hazard ratio.

During 618,582.6 person-years of follow-up, 27,817 cases of incident HS were identified (incidence rate 45.0 per 10^3^ person-years). Median follow-up was 4.0 years (interquartile range, 2.1–6.1). After adjustment for age, sex, center, year of the screening examination, season, alcohol consumption, smoking, physical activity, total energy intake, marital status, education level, depressive symptoms, history of diabetes, and history of hypertension (Table 2), multivariable-adjusted HRs (95% CIs) for incident HS comparing sleep durations of ≤5, 6, 8, and ≥9 hours with 7 hours were 1.19 (1.14–1.23), 1.07 (1.04–1.10), 0.98 (0.94–1.02), and 0.95 (0.87–1.03), respectively. After further adjustment for body mass index (BMI) (model 3), the association between short sleep duration and incident HS was attenuated but remained statistically significant. Results were similar if BMI was replaced by the waist circumference (model 4). In time-dependent analyses where change in sleep duration, sleep quality, and other covariates during follow-up were treated as a time-varying covariate, these results were similar. In the spline regression analyses, there was a dose-response relationship between sleep duration and the development of NAFLD (Figure 2).

**Table 2.** Hazard ratios^a^ (95% CI) of incident hepatic steatosis (regardless of fibrosis score) by sleep duration and subjective sleep quality

	Sleep duration (hr)					*P* for trend	Subjective sleep quality	
	≤5	6	7	8	≥9		Good	Poor
PY	78,095	204,972	216,532	97,316	21,668		498,010	120,573
Incident cases	4,128	10,567	9,326	3,244	552		22,982	4,835
Incidence density (/10^3^ PY)	52.9	51.6	43.1	33.3	25.5		46.1	40.1
Multivariable-adjusted HR								
Model 1	1.24 (1.19–1.28)	1.09 (1.06–1.12)	1.0	0.97 (0.94–1.01)	0.94 (0.86–1.03)	<0.001	1.0	1.07 (1.04–1.11)
Model 2	1.19 (1.14–1.23)	1.07 (1.04–1.10)	1.0	0.98 (0.94–1.02)	0.95 (0.87–1.03)	0.002	1.0	1.00 (0.97–1.04)
Model 3	1.07 (1.03–1.11)	1.02 (0.99–1.05)	1.0	0.99 (0.96–1.04)	1.00 (0.91–1.09)	0.001	1.0	1.05 (1.02–1.09)
Model 4	1.10 (1.05–1.14)	1.03 (1.00–1.06)	1.0	0.97 (0.93–1.01)	0.95 (0.87–1.04)	<0.001	1.0	1.03 (1.00–1.07)
Time-dependent model 1^b^	1.20 (1.15–1.25)	1.07 (1.04–1.10)	1.0	0.97 (0.93–1.01)	0.88 (0.80–0.97)	<0.001	1.0	0.97 (0.94–1.01)
Time-dependent model 2^b^	1.06 (1.02–1.10)	1.01 (0.99–1.04)	1.0	0.99 (0.95–1.03)	0.95 (0.86–1.04)	0.001	1.0	1.02 (0.99–1.06)
Time-dependent model 3^b^	1.16 (1.06–1.15)	1.03 (1.001–1.06)	1.0	0.98 (0.94–1.02)	0.91 (0.83–1.001)	<0.001	1.0	1.00 (0.96–1.03)

BMI, body mass index; CI, confidence interval; HR, hazard ratio; PY, person-years.

a Estimated from parametric proportional hazard models. Multivariable model 1 was adjusted for age and sex; model 2: model 1 plus adjustment for center, year of screening examination, season, alcohol consumption, smoking, physical activity, total energy intake, marital status, season, education level, depression, history of diabetes, and history of hypertension; model 3: model 2 plus adjustment for BMI; and model 4: model 2 plus adjustment for waist circumference.

b Estimated from parametric proportional hazard models with sleep duration, smoking, alcohol consumption, physical activity, total energy intake, marital status, season, depression, history of diabetes, and history of hypertension as time-dependent categorical variables and baseline age, sex, center, year of screening examination, education level as time-fixed variables: time-dependent model 2; time-dependent model 1 plus adjustment for BMI as time-varying variable; time-dependent model 3; time-dependent model 1 plus adjustment for waist circumference as time-varying variable.

During 680,986.1 person-years of follow-up, 1,471 cases of incident HS plus an intermediate/high FIB-4 were identified (incidence rate 2.2 per 10^3^ person-years). After adjustment for potential confounders, the multivariable-adjusted HRs (95% CI) for incident HS plus an intermediate/high FIB-4 comparing sleep durations of ≤ 5, 6, 8, and ≥ 9 hours with 7 hours (reference) were 1.30 (1.11–1.54), 1.14 (1.01–1.29), 1.11 (0.93–1.33), and 1.08 (0.71–1.63), respectively. Results were similar based on the NFS. After further adjustment for BMI (model 3), the association between short sleep duration and incident HS plus intermediate/high fibrosis markers remained significant. By contrast, poor sleep quality was not significantly associated with the risk of either incident HS or HS plus intermediate/high fibrosis markers (Tables 2 and 3). All these associations were consistently observed in both men and women without significant interaction by sex.

**Table 3.** Hazard ratios^a^ (95% confidence interval) of incident hepatic steatosis plus intermediate/high probability of advanced fibrosis by sleep duration and subjective sleep quality

	Sleep duration (hr)					*P* for trend	Subjective sleep quality	
	≤5	6	7	8	≥9		Good	Poor
HS + intermediate/high based on FIB-4								
PY	87,425	229,519	237,443	103,907	22,692		550,419	130,567
Incident cases	232	591	461	163	24		1,242	229
Incidence density (/10^3^ PY)	2.7	2.6	1.9	1.6	1.1		2.3	1.8
Multivariable-adjusted HR								
Model 1	1.37 (1.17–1.61)	1.16 (1.03–1.31)	1.0	1.11 (0.93–1.33)	1.06 (0.70–1.60)	0.002	1.0	1.08 (0.94–1.25)
Model 2	1.30 (1.11–1.54)	1.14 (1.01–1.29)	1.0	1.11 (0.93–1.33)	1.08 (0.71–1.63)	0.021	1.0	0.97 (0.84–1.13)
Model 3	1.19 (1.01–1.40)	1.08 (0.95–1.22)	1.0	1.14 (0.96–1.37)	1.14 (0.75–1.72)	0.392	1.0	1.02 (0.88–1.19)
Model 4	1.25 (1.05–1.49)	1.08 (0.94–1.24)	1.0	1.03 (0.84–1.27)	0.99 (0.62–1.57)	0.038	1.0	0.96 (0.81–1.13)
Time-dependent model 1^b^	1.21 (1.03–1.43)	1.03 (0.91–1.17)	1.0	0.86 (0.71–1.05)	1.03 (0.67–1.56)	0.006	1.0	1.03 (0.89–1.19)
Time-dependent model 2^b^	1.06 (0.91–1.25)	0.97 (0.86–1.10)	1.0	0.89 (0.73–1.08)	1.08 (0.71–1.64)	0.339	1.0	1.08 (0.93–1.25)
Time-dependent model 3^b^	1.09 (0.92–1.28)	0.99 (0.87–1.12)	1.0	0.86 (0.70–1.05)	1.03 (0.68–1.56)	0.139	1.0	1.05 (0.90–1.22)
HS + intermediate/high based on NFS								
PY	87,081	228,463	236,841	103,769	22,657		548,535	130,274
Incident cases	339	339	716	215	39		1911	353
Incidence density (/10^3^ PY)	3.9	4.2	3.0	2.1	1.7		3.5	2.7
Multivariable-adjusted HR								
Model 1	1.30 (1.14–1.48)	1.22 (1.11–1.35)	1.0	0.93 (0.80–1.09)	1.10 (0.80–1.53)	<0.001	1.0	1.07 (0.95–1.20)
Model 2	1.26 (1.10–1.44)	1.21 (1.10–1.33)	1.0	0.93 (0.80–1.09)	1.11 (0.80–1.53)	<0.001	1.0	1.01 (0.90–1.14)
Model 4	1.15 (1.00–1.34)	1.13 (1.01–1.26)	1.0	0.89 (0.74–1.06)	1.15 (0.80–1.63)	0.005	1.0	1.01 (0.88–1.15)
Time-dependent model 1^b^	1.20 (1.05–1.37)	1.16 (1.05–1.28)	1.0	0.86 (0.73–1.01)	0.95 (0.67–1.35)	<0.001	1.0	0.99 (0.87–1.12)
Time-dependent model 3^b^	1.01 (0.88–1.16)	1.07 (0.97–1.18)	1.0	0.86 (0.73–1.01)	0.97 (0.69–1.38)	0.065	1.0	1.02 (0.90–1.16)

BMI, body mass index; FIB-4, fibrosis-4 index; NFS, NAFLD fibrosis score; PY, person-years.

a Estimated from parametric proportional hazard models. Multivariable model 1 was adjusted for age and sex; model 2: model 1 plus adjustment for center, year of screening examination, season, alcohol consumption, smoking, physical activity, total energy intake, marital status, season, education level, depression, history of diabetes (only for FIB-4), and history of hypertension; model 3: model 2 plus adjustment for BMI; and model 4: model 2 plus adjustment for waist circumference.

b Estimated from parametric proportional hazard models with sleep duration, smoking, alcohol consumption, physical activity, total energy intake, marital status, depression, history of diabetes, and history of hypertension as time-dependent categorical variables and baseline age, sex, center, year of screening examination, education level as time-fixed variables: time-dependent model 2; time-dependent model 1 plus adjustment for BMI as time-varying variable; time-dependent model 3; time-dependent model 1 plus adjustment for waist circumference as time-varying variable.

The associations between sleep duration and risk of HS were similarly observed in those with or without poor sleep quality (*P* for interaction = 0.743), whereas the association between sleep duration and HS plus intermediate/high fibrosis markers significantly differed by sleep quality (Table 4). There was an inverse association between sleep duration and HS plus an intermediate/high FIB-4 in individuals with good sleep quality, but it tended to be U-shaped in those with poor sleep quality. These associations were similarly observed in the analysis using NFS instead of FIB-4.

**Table 4.** Hazard ratios^a^ (95% CI) of incident HS without or with intermediate/high probability of advanced fibrosis by sleep duration among subjects with and without poor sleep quality

	Sleep duration (hr)					*P* for trend	*P* for
	≤5	6	7	8	≥9		Interaction
HS regardless of fibrosis score							
Sleep quality							0.743
Good (N = 114,577)	1.20 (1.15–1.25)	1.08 (1.04–1.11)	1.0	0.98 (0.94–1.03)	0.94 (0.86–1.03)	<0.001	
Poor (N = 28,729)	1.13 (1.05–1.23)	1.05 (0.97–1.13)	1.0	0.94 (0.83–1.08)	0.99 (0.78–1.26)	<0.001	
HS + intermediate/high FIB-4							
Sleep quality							0.047
Good (N = 114,577)	1.31 (1.08–1.58)	1.11 (0.97–1.26)	1.0	1.12 (0.93–1.35)	0.85 (0.52–1.38)	0.033	
Poor (N = 28,729)	1.45 (0.97–2.15)	1.41 (0.96–2.08)	1.0	0.95 (0.46–1.98)	3.33 (1.48–7.51)	0.365	
HS + intermediate/high NFS							
Sleep quality							0.068
Good (N = 114,577)	1.28 (1.10–1.49)	1.18 (1.07–1.31)	1.0	0.90 (0.77–1.06)	0.94 (0.65–1.36)	<0.001	
Poor (N = 28,729)	1.36 (0.99–1.88)	1.44 (1.06–1.97)	1.0	1.34 (0.80–2.23)	2.72 (1.34–5.51)	0.684	

*P for quadratic term* = 0.001 for the association between sleep duration and incident HS; *P for quadratic term* = 0.08 for the association between sleep duration and incident HS plus intermediate/high FIB-4; and *P for quadratic term* = 0.192 for the association between sleep duration and incident HS plus intermediate/high NFS among participants with poor sleep quality.

CI, confidence interval; FIB-4, fibrosis-4 index; HR, hazard ratio; HS, hepatic steatosis; NFS, NAFLD fibrosis score.

a Estimated from parametric proportional hazard models. Multivariable model was adjusted for age, sex, center, year of screening examination, season, alcohol consumption, smoking, physical activity, total energy intake, marital status, season, education level, depression, history of diabetes (not for HS + intermediate/high NFS), and history of hypertension.

Because obesity is closely associated with HS and sleep duration is also related to obesity, we performed analyses among nonobese individuals to address residual confounding factors because of obesity. The associations between sleep duration, HS, and HS plus an intermediate/high FIB-4 were similar in nonobese individuals with a BMI of <25 kg/m^2^. Specifically, after adjustment for age, sex, center, year of screening examination, alcohol consumption, smoking, physical activity, season, total energy intake, marital status, education level, depression, and history of hypertension, multivariable-adjusted HRs (95% CIs) for incident HS regardless of fibrosis score comparing sleep durations of ≤5, 6, 8, and ≥ 9 hours with 7 hours were 1.17 (1.12–1.22), 1.07 (1.04–1.11), 0.98 (0.93–1.02), and 0.88 (0.80–0.97), respectively. The corresponding HRs (95% CIs) for HS plus an intermediate/high FIB-4 were 1.38 (1.05–1.79), 1.20 (0.98–1.47), 1.13 (0.86–1.49), and 1.20 (0.68–2.11), respectively.

## DISCUSSION

In this large-scale prospective cohort study of relatively young adults with a median age of 36.6 years, a median follow-up of over 4 years and the availability of repeated measurements of sleep habits, NAFLD status, and other covariates, short sleep duration was found to be independently associated with an increased risk of developing NAFLD both with and without an intermediate/high fibrosis score. These associations between short sleep duration and increased risk of developing NAFLD were attenuated after adjustment for either BMI or waist circumference, but still remained significant. In addition, the associations remained significant after adjustment for changes in sleep duration and other confounders over time (as time-varying covariates in the time-dependent models). Interestingly, sleep quality was not significantly associated with the risk of NAFLD, and the association between sleep duration and HS did not significantly differ by sleep quality. However, the association between sleep duration and HS plus intermediate/high FIB-4 significantly differed by sleep quality. In individuals with good sleep quality, sleep duration was inversely associated with HS plus an intermediate/high FIB-4 in a dose-response manner, whereas both short and long sleep durations were associated with an increased risk of HS plus an intermediate/high FIB-4, showing a U-shaped association in those with poor sleep quality. The reason for the increased risk of HS plus an intermediate/high FIB-4 level among individuals with long sleep duration in those with poor sleep quality, and the decreased risk in those with good sleep quality, is not fully understood. Compared with individuals with normal sleep duration, those with long sleep duration have been reported as having a higher risk of obstructive sleep apnea and insomnia symptoms, such as increased sleep fragmentation, wake after sleep onset, and sleep latency (26). In addition, habitually long sleep duration has been reported to be associated with poor physical and mental health status (27,28); thus, excessively long sleep duration may be an indicator of coping with and compensating for this poor sleep quality and other unmeasured features of poor health status (26–28).

A meta-analysis including 5 cross-sectional studies and 1 cohort study found a small but significantly increased risk of NAFLD among individuals with a short sleep duration compared with those with a longer sleep duration (14). By contrast, another meta-analysis of 6 cross-sectional studies and 2 cohort studies found that neither short nor long sleep duration was related to NAFLD risk (15). However, these meta-analyses were both limited in that they included only a few cohort studies; the former only included 1 cohort study (14) and the latter 2 cohort studies (15).

An earlier cohort study of 2,133 middle-aged Japanese patients showed an association between short sleep duration and reduced risk of NAFLD (17), whereas a recent cohort study of 12,306 Japanese adults reported a significant association between short sleep duration and increased risk of NAFLD (19). A cohort study of 5,427 Korean adults reported that long sleep duration was associated with an increased incidence of NAFLD, based on the NAFLD scores rather than on ultrasonography (29). Finally, a different cohort study of 8,965 Chinese subjects with a mean age of 61.6 years demonstrated a positive association between long sleep duration (8–9 hr/d) and new-onset NAFLD (16). However, this study was limited in that it only included a very small number of subjects in the short sleep duration category (only 96 subjects with a sleep duration of <6 hours) (16). Also, the prevalence of sleep disorders increases with age, and approximately 50% of the elderly have sleep problems (30). None of previous studies evaluated the impact of sleep quality as either the main exposure or effect modifier on the risk of incident NAFLD.

Several plausible mechanisms may explain the association between sleep and NAFLD. Sleep deprivation may increase the ghrelin but decrease leptin levels, causing a rise in appetite (31), subsequently resulting in weight gain and obesity, which is a strong risk factor for NAFLD (1). In our study, adjustment for either BMI or waist circumference attenuated the relationship between sleep duration and incident HS or HS plus an intermediate/high FIB-4, but the results remained statistically significant. Furthermore, this association was observed in nonobese individuals with a BMI < 25 kg/m^2^. Therefore, the association between sleep duration and NAFLD seems to not be fully explained by obesity. However, given that the association between sleep duration and NAFLD risk was attenuated by approximately 14% after adjustment for BMI or waist circumference, the role of overall and abdominal obesity is likely to contribute to the association between sleep duration and NAFLD risk. Studies suggest that sleep deprivation and sleep disturbance can decrease insulin sensitivity (10,32), a key pathogenic mechanism of NAFLD. Second, sleep deprivation provokes proinflammatory activity (e.g., increased IL-6 or tumor necrosis factor—alpha [32, 33]), which can induce inflammation, another mechanism of NAFLD. Furthermore, melatonin is known to function as a strong antioxidant, and low levels of melatonin may influence liver disease (34). In support of the beneficial effect of melatonin, a randomized controlled trial of 100 patients with histologically proven NAFLD showed a beneficial effect of melatonin treatment on liver enzyme levels (35).

A major strength of our study is that we have shown the effect of sleep duration and sleep quality on NAFLD and its severity using a large-scale cohort. In addition, the availability of repeated measurements of sleep habits, NAFLD status, and other covariates enabled us to take into account the effects of changes in these variables as time-varying covariates. Furthermore, our findings are derived from a relatively young population less likely to be affected by survivor bias or bias related to comorbidities. However, age is an important risk factor for NAFLD and its progression (36,37). Therefore, our findings derived from young adults may not be generalizable to older populations. However, recent studies suggest that the prevalence of HS and fibrosis is increasing in younger populations (38,39). The results of our study emphasize the importance of starting lifestyle modifications at an early age to prevent progression of the disease. Currently, the risk factors for lean NAFLD are not fully understood (40), despite an increase in its prevalence, especially in Asians (41). Importantly, our study findings suggest that short sleep duration could be a risk factor for lean NAFLD because we also know that short sleep duration is associated with an increased risk of NAFLD in nonobese subjects. The effect size was comparably small. However, we emphasize that NAFLD is one of the most common liver diseases worldwide and sleep shortage is common, with an estimated prevalence of more than 20% (9,42). In the absence of approved medication for NAFLD, healthy lifestyle adoption, which also includes better quality and adequate sleep duration, continues to be at the center of primary and secondary prevention of this disease.

There were several limitations to our study. First, sleep duration was self-reported in the PSQI questionnaire, which has been reported to show a moderate correlation with objectively measured sleep duration (43). In a study of 112 volunteers, consisting of a group aged 18–32 years (n = 59) and an older group aged 59–75 years (n = 53), the global and component scores of the PSQI correlated well with sleep diary variables and Center for Epidemiologic studies depression scale scores (44). By contrast, the PSQI score did not correlate with sleep variables measured using actigraph accelerometers in the whole group, while significant correlations were only observed between the PSQI sleep duration component and total sleep time in the younger group (44). Further studies with objective and subjective sleep measures are required to confirm the relationship between sleep quantity and quality and NAFLD risk. Second, a histologic assessment of the liver was not performed. However, abdominal ultrasonography is widely used in large cohort studies, as a measure to diagnose HS with acceptable diagnostic accuracy for the detection of fatty liver (45). Although a high FIB-4 or NFS is a validated proxy measure of high probability of advanced liver fibrosis (24), it is possible that some subjects with intermediate FIB-4 score or NFS do not have liver fibrosis. Finally, our study population comprised relatively young and middle-aged Koreans, possibly limiting the generalizability of our findings to other age groups, populations with a higher prevalence of comorbidities, or other ethnic groups.

In conclusion, our results show that short sleep duration was associated with an increased risk of incident NAFLD, both with and without intermediate/high fibrosis scores at follow-up. We suggest that interventional studies that modify sleep duration are necessary to test whether there is a beneficial effect of ameliorating sleep deprivation on the risk of NAFLD.

## CONFLICTS OF INTEREST

**Guarantor of the article:** Seungho Ryu, MD, PhD, and Yoosoo Chang, MD, PhD.

**Specific author contributions:** Y.J.U: drafting of the manuscript and critical revision of the manuscript. Y.C: study concept and design; acquisition of data; interpretation of data; drafting of the manuscript; and critical revision of the manuscript. H.S.J: acquisition of data; interpretation of data; and critical revision of the manuscript. I.Y.C: interpretation of data and critical revision of the manuscript. J.H.S: technical or material support and study supervision. H.S: technical or material support and study supervision. S.H.W: interpretation of data and critical revision of the manuscript. C.D.B: interpretation of data and critical revision of the manuscript. S.R: study concept and design; acquisition of data; analysis and interpretation of data; and critical revision of the manuscript.

**Financial support:** None to report.

**Potential competing interests:** None to report.

**Study Highlights**

**WHAT IS KNOWN**

✓ Nonalcoholic fatty liver disease (NAFLD) is associated with the risk of cardiometabolic disease.
✓ Inadequate sleep duration is related to obesity, metabolic syndrome, and cardiovascular disease.
✓ The cohort studies on the relationship of sleep duration and NAFLD show conflicting results.

**WHAT IS NEW HERE**

✓ Short sleep duration is a risk factor for developing NAFLD with or without fibrosis.
✓ Body mass index only partially mediated the association between sleep duration and NAFLD.
✓ Changes in sleep duration as a time-varying covariate produced similar results.

## Supplementary Material

SUPPLEMENTARY MATERIAL
