# Supplementary material for: Sleep Duration, Sleep Quality, and the Development of Nonalcoholic Fatty Liver Disease: A Cohort Study
Source: Clin Transl Gastroenterol. 2021 Oct 19;12(10):e00417. doi: 10.14309/ctg.0000000000000417 (PMC8528229; doi:10.14309/ctg.0000000000000417)
Supplement: SUPPLEMENTARY MATERIAL [file ct9-12-e00417-s001.docx]

**SUPPLEMENTARY MATERIALS**

***S1. Measurement***

Pittsburgh Sleep Quality Index (PSQI), 19-item self-administered questionnaire, at baseline and during the follow-up sessions was used to assess sleep duration and quatliy.(1) It consists of seven components, including subjective sleep quality, sleep latency, sleep duration, habitual sleep efficiency, sleep disturbances, use of sleeping medication, and daytime function. Each component score ranges from 0 (best) to 3 (worst sleep properties), and the PSQI score is calculated as the sum of each component score to generate an overall score. In one of the PSQI items, the subjects were asked to report the hours of actual asleep at night in a typical 24 h period over the previous month. Sleep duration was obtained by rounding values of 30 min or more up to the nearest hour and rounding values less than 30 min down to the nearest hour. Sleep quality was categorized into two groups (good: PSQI score <6, poor: PSQI score ≥6) (1). Sleep duration were grouped into ≤5, 6, 7, 8, and ≥9 h. Since 7 hours per day or more for adults are considered appropriate to maintain ideal health,(2) 7 hours was chosen as the reference and shorter duration was defined as short sleep duration.

Depressive symptoms were assessed using the Korean version of the Center for Epidemiologic Studies Depression (CES-D) scale and were categorized as a CES-D score of <16 and ≥16.(3, 4)

Physical activity levels were assessed using the validated Korean version of the International Physical Activity Questionnaire Short Form.(5, 6) Health-enhancing physical activity (HEPA) was defined as follows: (1) vigorous activity ≥3 days/week with ≥1,500 accumulated metabolic equivalent (MET)-minutes/week, or (2) a combination of walking, moderate- or vigorous-intensity activities for 7 days accumulating to ≥3,000 MET-min/week.

Sitting blood pressure (BP), height, weight, and waist circumference were measured by trained nurses. Hypertension was defined as a systolic blood pressure ≥140 mmHg, diastolic blood pressure ≥90 mmHg, or current use of antihypertensive medication.

Blood and urine specimens were collected after at least 10 h of fasting. Fasting blood sample measurements included total cholesterol, low density lipoprotein-cholesterol (LDL-C), high density lipoprotein-cholesterol (HDL-C), triglycerides, AST, ALT, gamma-glutamyl transferase (GGT), glucose, uric acid, hsCRP, albumin, and platelet count. The homeostatic model assessment of insulin resistance (HOMA-IR) index was calculated as follows: fasting blood insulin (mU/mL) × fasting blood glucose (mmol/L)/22.5.

***S2. Assessment of hepatic steatosis and non-invasive fibrosis score***

The diagnosis of hepatic steatosis was determined using standard criteria, including the presence of a diffuse increase in fine echoes in the liver parenchyma compared with the kidney or spleen parenchyma, deep beam attenuation, and bright vessel walls.(7) Inter-observer and intra-observer reliability values for HS diagnoses were moderate to substantial (kappa statistic of 0.74) (8, 9) and excellent (kappa statistic of 0.94), respectively.(10)

The FIB-4 index was calculated using the following formula: FIB-4 = [age (years) × AST (U/L)] / [platelet count (×10^9^/L) × ALT (U/L)^1/2^]. The subjects were classified into three groups, reflecting the probability of advanced fibrosis based on the FIB-4 score: low (FIB-4 <1.30), intermediate (FIB-4 1.30-2.66), and high (FIB-4 ≥2.67).(11) NFS was calculated according to the following published formula: NFS = -1.675 + 0.037 × age (years) + 0.094 × BMI (kg/m^2^) + 1.13 × impaired fasting glycemia or diabetes (yes = 1, no = 0) + 0.99 × AST/ALT ratio - 0.013 × platelet (×10^9^/L) - 0.66 × albumin (g/dL).(12) Subjects were also categorized into three groups reflecting the probability of advanced fibrosis based on the NFS: high (NFS >0.676), intermediate (NFS: 0.676 to -1.455), and low (NFS < -1.455).(12)

***S3. Statistical modeling for data analysis***

The models were initially adjusted for age and sex and then adjusted for study center (Seoul, Suwon), year of the screening exam, season (spring, summer, fall, and winter), smoking (never, past, current, or unknown), alcohol intake (none, < 10, or ≥ 10 g/day, or unknown), physical activity (inactive, minimally active, health-enhancing physical activity (HEPA), or unknown), CES-D (<16, ≥ 16, or unknown), education level (< community college graduate, ≥ community college graduate, or unknown), total energy intake, history of diabetes, history of hypertension, and history of CVD (Model 2). Next, we sought to examine whether the relationship between sleep duration and development of the primary endpoints was mediated by body mass index (BMI) as a continuous variable (Model 3) on a priori grounds.

We evaluated the mediation effect of BMI on the association between sleep duration and risk of HS or HS plus an intermediate/high probability of liver fibrosis (Model 4) if the BMI met the three criteria for being a potential mediator as follows: 1) sleep duration was associated with BMI, 2) BMI was significantly associated with the incident endpoint when sleep duration was included in the model, and 3) the addition of BMI to the model attenuated the association between sleep duration and incident HS. To explore the role of central obesity, these analyses were repeated when BMI was replaced by waist circumference (Model 4).

To further explore the shape of the dose-response relationship of sleep duration with the development of NAFLD, restricted cubic splines with knots were used at the 5^th^, 27.5^th^, 50^th^, 72.5^th^, and 95^th^ percentiles of sleep duration. We then evaluated whether the associations between sleep duration and the risk of NAFLD differed by sleep quality. The interactions between sleep quality and sleep duration categories on the risk of NAFLD were tested using likelihood ratio tests. These tests were used to compare the models with and without multiplicative interaction terms.

**References**

1. Buysse DJ, Reynolds CF, 3rd, Monk TH, et al. The Pittsburgh Sleep Quality Index: a new instrument for psychiatric practice and research. Psychiatry Res 1989;28:193-213.

2. Hirshkowitz M, Whiton K, Albert SM, et al. National Sleep Foundation's sleep time duration recommendations: methodology and results summary. Sleep Health 2015;1:40-43.

3. Radloff LS. The CES-D scale: a self-report depression scale for research in the general population. Appl Psychol Meas 1977;1:385-401.

4. Cho MJ, Kim KH. Diagnostic validity of the CES-D (Korean version) in the assessment of DSM-III R major depression. J Korean Neuropsychiatr Assoc 1993;32:381-399.

5. Chun MY. Validity and reliability of korean version of international physical activity questionnaire short form in the elderly. Korean J Fam Med 2012;33:144-51.

6. Craig CL, Marshall AL, Sjostrom M, et al. International physical activity questionnaire: 12-country reliability and validity. Med Sci Sports Exerc 2003;35:1381-95.

7. Mathiesen UL, Franzen LE, Aselius H, et al. Increased liver echogenicity at ultrasound examination reflects degree of steatosis but not of fibrosis in asymptomatic patients with mild/moderate abnormalities of liver transaminases. Dig Liver Dis 2002;34:516-22.

8. McHugh ML. Interrater reliability: the kappa statistic. Biochem Med (Zagreb) 2012;22:276-82.

9. Sim J, Wright CC. The kappa statistic in reliability studies: use, interpretation, and sample size requirements. Phys Ther 2005;85:257-68.

10. Chang Y, Ryu S, Sung KC, et al. Alcoholic and non-alcoholic fatty liver disease and associations with coronary artery calcification: evidence from the Kangbuk Samsung Health Study. Gut 2019;68:1667-1675.

11. Shah AG, Lydecker A, Murray K, et al. Comparison of noninvasive markers of fibrosis in patients with nonalcoholic fatty liver disease. Clin Gastroenterol Hepatol 2009;7:1104-12.

12. Angulo P, Hui JM, Marchesini G, et al. The NAFLD fibrosis score: a noninvasive system that identifies liver fibrosis in patients with NAFLD. Hepatology 2007;45:846-54.
